# Association of Reduced Nicotine Content Cigarettes With Smoking Behaviors and Biomarkers of Exposure Among Slow and Fast Nicotine Metabolizers: A Nonrandomized Clinical Trial

**DOI:** 10.1001/jamanetworkopen.2018.1346

**Published:** 2018-08-24

**Authors:** Melissa Mercincavage, Kirsten Lochbuehler, E. Paul Wileyto, Neal L. Benowitz, Rachel F. Tyndale, Caryn Lerman, Andrew A. Strasser

**Affiliations:** 1 Center for Interdisciplinary Research on Nicotine Addiction, Department of Psychiatry, Perelman School of Medicine, University of Pennsylvania, Philadelphia; 2 Department of Biostatistics & Epidemiology, Perelman School of Medicine, University of Pennsylvania, Philadelphia; 3 Department of Medicine, University of California, San Francisco; 4 Department of Bioengineering & Therapeutic Sciences, University of California, San Francisco; 5 Centre for Addiction and Mental Health, Department of Pharmacology and Toxicology, University of Toronto, Toronto, Ontario, Canada; 6 Division of Brain & Therapeutics, Department of Psychiatry, University of Toronto, Toronto, Ontario, Canada; 7 Abramson Cancer Center, University of Pennsylvania, Philadelphia

## Abstract

**Importance:**

The US Food and Drug Administration (FDA) has announced its intention to reduce the nicotine content in combustible cigarettes but must base regulation on public health benefits. Fast nicotine metabolizers may be at risk for increased smoking following a national nicotine reduction policy. We hypothesized that using reduced nicotine content (RNC) cigarettes would be associated with increases in smoking behaviors and exposure among smokers with a fast—but not slow—nicotine-metabolite ratio (NMR).

**Objectives:**

To examine the association of RNC cigarettes with smoking behaviors and biomarkers of exposure and to compare these associations in fast and slow metabolizers of nicotine based on the NMR.

**Design, Setting, and Participants:**

A 35-day, 3-period, within-participant nonrandomized clinical trial was conducted at an academic medical center in Philadelphia, Pennsylvania. A 5-day baseline period using the smokers’ preferred brand of cigarettes was followed by 2 consecutive 15-day periods using free investigational RNC cigarettes. A total of 100 daily, non–treatment-seeking, nonmenthol cigarette smokers (59 fast, 41 slow metabolizers) were recruited from December 24, 2013, to December 2, 2015. Data analysis was performed from December 12, 2016, to January 3, 2018.

**Interventions:**

Two 15-day periods using cigarettes containing 5.2 mg (RNC1) and 1.3 mg (RNC2) of nicotine per gram of tobacco.

**Main Outcomes and Measures:**

Smoking behaviors (number of cigarettes per day [CPD], total puff volume) and biomarkers of exposure (carbon monoxide [CO], urine total nicotine equivalents [TNE], and 4-[methylnitrosamino]-1-[3-pyridyl]-1-butanol [NNAL]).

**Results:**

Smokers (73 [73.0%] men; 74 [74.0%] white; mean [SD] age, 43.02 [12.13] years; mean [SD] CPD, 17.31 [5.72]) consumed 2.62 (95% CI, 1.54-3.70) more CPD during the RNC1 period vs their preferred brand during baseline (*P* < .001) and approximated baseline CPD during the RNC2 period (mean difference, 0.96 [95% CI, −0.36 to 2.28]; *P* = .24). Additional outcome measures were lower during both RNC periods vs baseline (total puff volume, mean [95% CI]: RNC1, 537 mL [95% CI, 479-595 mL]; RNC2, 598 mL [95% CI, 547-649 mL] vs baseline, 744 mL [95% CI, 681-806 mL]; TNE, mean [95% CI]: RNC1, 30.9 nmoL/mg creatinine [95% CI, 26.0-36.6 nmoL/mg]; RNC2, 22.8 nmoL/mg creatinine [95% CI, 17.8-29.0 nmoL/mg] vs baseline, 54.6 nmoL/mg creatinine [95% CI, 48.1-62.1 nmoL/mg]; and NNAL, mean [95% CI]: RNC1, 229 pg/mg creatinine [95% CI, 189-277 pg/mg]; RNC2, 190 pg/mg creatinine [95% CI, 157-231 pg/mg] vs baseline, 280 pg/mg creatinine [95% CI, 231-339 pg/mg]; all *P* < .001). Carbon monoxide measures were similar across study periods (CO boost [SD], RNC1, 4.6 ppm [4.1-5.1 ppm]; RNC2, 4.2 ppm [3.7-4.6 ppm]; and baseline, 4.4 ppm [3.8-4.9 ppm]). The RNC cigarette associations did not differ by NMR.

**Conclusions and Relevance:**

Both RNC cigarettes were associated with decreased puffing and urinary biomarker exposure but not with decreased daily cigarette consumption or CO levels. The NMR did not moderate associations at the nicotine levels tested, suggesting that fast metabolizers may not be at greater risk of increasing use or exposure from these products should the FDA mandate an RNC standard for cigarettes.

**Trial Registration:**

ClinicalTrials.gov identifier: NCT01898507

## Key Points

**Question:**

Would federally mandated nicotine reduction in cigarettes adversely affect smokers with fast nicotine metabolism?

**Findings:**

In this nonrandomized clinical trial of 100 adult smokers, use of reduced nicotine content cigarettes for two 15-day periods was associated with decreased puffing behaviors and urinary tobacco biomarker exposure relative to smokers’ preferred brand but not with decreased daily cigarette consumption or carbon monoxide levels. These associations did not differ significantly between slow and fast nicotine metabolizers.

**Meaning:**

This study suggests that fast nicotine metabolizers may not be at greater risk of being negatively affected should the US Food and Drug Administration mandate a reduced nicotine product standard similar to the nicotine levels tested.

## Teaser

This nonrandomized clinical trial of daily cigarette smokers evaluates the association of use of reduced nicotine content cigarettes with smoking behaviors and biomarkers of exposure by smokers with fast vs slow nicotine metabolism.

## Introduction

On July 28, 2017, the US Food and Drug Administration (FDA) announced its intent to decrease the addictiveness of combustible cigarettes by reducing their nicotine content.^1,2^ This regulatory strategy is authorized under the 2009 Family Smoking Prevention and Tobacco Control Act.^3^ Emerging data suggest that reduced nicotine content (RNC) cigarettes decrease nicotine and tobacco-specific nitrosamine exposure, dependence, and daily cigarette consumption^4,5,6,7,8,9,10,11,12^ without causing long-term compensatory behaviors.^13^ Further, these products may promote cessation among treatment-seeking and non–treatment-seeking smokers.^9,11,14,15^

Because the Family Smoking Prevention and Tobacco Control Act does not permit the FDA to eliminate nicotine from cigarettes,^3^ a question remains as to what level the nicotine content should be reduced. The results of switching to RNC cigarettes are not consistent across all RNC levels or all use and exposure measures.^4,5,6,7,8,9,10,11,12^ For example, cigarettes with modest reductions in nicotine content decrease puffing behavior yet increase cigarette consumption,^5,7,12^ and greater reductions decrease nicotine exposure but not carbon monoxide (CO) levels.^12,16^ These complex actions emphasize the need for studies that use rigorous behavioral assessments of use and biomarkers of exposure at different nicotine levels.

Because the Family Smoking Prevention and Tobacco Control Act states that FDA regulations must yield public health benefits, another critical question is whether nicotine reduction would negatively affect subgroups of smokers.^17^ Studies have evaluated sex,^18,19,20^ cannabis use,^21^ and mental health issues^22,23^ as possible moderators of RNC cigarette effects to identify at-risk subgroups, with only sex influencing responses. Another factor requiring examination is rate of nicotine metabolism, assessed using the nicotine-metabolite ratio (NMR; the ratio of *trans*-3′-hydroxycotinine to cotinine^24,25,26^), a heritable,^27^ stable^28^ trait that also reflects environmental and hormonal factors. For example, African American individuals have, on average, slower nicotine metabolism and higher cotinine levels than white individuals,^29,30^ and use of estrogen-based oral contraceptives accelerates nicotine metabolism.^31^ The rate of nicotine metabolism alters smoking behaviors and toxicant exposure when conventional cigarettes are used. Compared with slow metabolizers, fast metabolizers puff their cigarettes more intensely, smoke more cigarettes and extract more nicotine per day, have higher dependence scores and lung cancer rates, and have lower cessation likelihood with transdermal nicotine compared with varenicline use.^31,32,33,34,35,36,37,38,39^ Thus, nicotine reduction could adversely affect fast metabolizers if they increase their smoking of RNC cigarettes to compensate for lower nicotine availability, potentially increasing exposure to harmful tobacco constituents.

Two studies have evaluated the NMR as a moderator of RNC cigarette effects,^40,41^ although others^7^ have included the NMR as an analysis covariate. In an acute laboratory study^40^ of overnight-abstinent young adult smokers exposed to 4 RNC cigarette levels, the NMR moderated associations with craving and withdrawal relief but not puffing behavior. In addition, the NMR did not affect smoking behaviors or exposure during a longitudinal study^41^ of adult smokers given progressively reduced RNC cigarettes over 6 months. Because these studies had varied design elements (eg, populations, single laboratory exposure vs extended real-world product use), further research is needed to better understand how the NMR may affect RNC cigarette use.

The present study aimed to (1) thoroughly characterize associations of RNC cigarette levels with smoking behaviors and biomarkers of exposure and (2) evaluate the NMR as a potential moderator of these associations. We extend prior research by using multiple behavior and exposure measures, assessed repeatedly over extended use periods. Based on the largest randomized clinical RNC cigarette trial,^7^ we hypothesized that, compared with baseline, RNC cigarettes would be associated with decreased puffing behavior and urinary biomarkers but not CO levels. We also hypothesized that RNC cigarettes with moderate and very low nicotine content would be associated with increased and decreased daily cigarette consumption, respectively. Finally, based on previous work,^34^ we hypothesized that the NMR would moderate RNC cigarette associations such that RNC cigarettes would be associated with increases in smoking behaviors and biomarkers of exposure among fast metabolizers.

## Methods

### Design Overview

**Figure 1.**
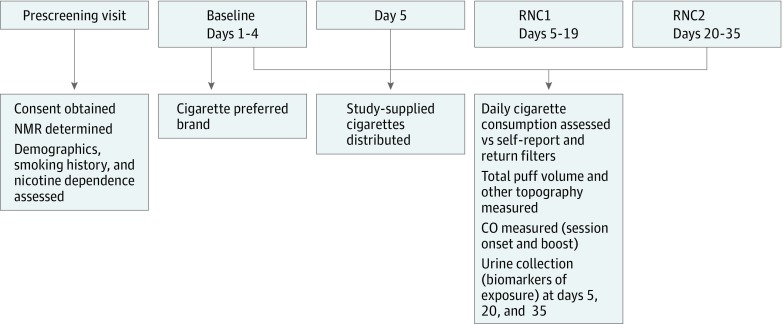
Study Design Overview CO indicates carbon monoxide; NMR, nicotine-metabolite ratio; and RNC, reduced nicotine content of 5.2 mg (RNC1) and 1.3 mg (RNC2) of nicotine per gram of tobacco.

After attending a preliminary screening session at an academic medical center to provide a blood sample to determine eligibility based on NMR (as discussed in the Measures section), 100 adult smokers participated in a 35-day, 3-period, within-participant, laboratory-based, nonrandomized clinical trial. After a 5-day baseline period of smoking their preferred brand of cigarettes, participants were assigned to use cigarettes containing 5.2 mg of nicotine per gram of tobacco (mg/g) followed by 1.3-mg/g cigarettes for 2 consecutive 15-day periods; in-person laboratory visits occurred every 5 days (Figure 1).^12,42^ Participants provided written informed consent and received financial compensation for completing all procedures during eight 2-hour sessions. The University of Pennsylvania Institutional Review Board approved all procedures. This study followed the Consolidated Standards of Reporting Trials (CONSORT) reporting guideline. The trial protocol is available in the Supplement.

### Participants

We recruited daily, non–treatment-seeking smokers interested in trying “a low nicotine cigarette product” from the Philadelphia, Pennsylvania, area from December 24, 2013, to December 2, 2015, using print and digital advertising and through contacting former participants. Prospective participants attended a preliminary screening session to complete demographic and smoking history questionnaires, provide a blood sample, and verify eligibility.

Eligible participants were aged 21 to 65 years, fluent in English, capable of providing informed consent, and reported smoking 10 or more filtered, nonmenthol cigarettes per day (CPD) for the past 5 years. We excluded individuals who reported daily use of other nicotine-containing products (ie, cigars, chewing tobacco, nicotine patch or gum, or electronic cigarettes); reported 25 or more alcohol-containing drinks per week or medication affecting nicotine biotransformation within the past 14 days; were enrolled or planned to enroll in a cessation program; reported a serious or unstable disease or history of substance abuse (excluding nicotine dependence) in the past year; were pregnant and/or lactating; had a history or current diagnosis of chronic obstructive pulmonary disease, stroke, myocardial infarction, psychosis, depression, bipolar disorder, mania, or schizophrenia; produced a positive urine drug screen result for cocaine, opiates, or methamphetamine; or provided an initial breath CO reading lower than 10 ppm.

### Procedures

Participants who met eligibility criteria at the preliminary screening session returned for study day 1 (Figure 1). They smoked 3 cigarettes, with 45 minutes between each one: the first cigarette standardized recency of smoking across participants, and the latter 2 were smoked through topography equipment (Clinical Research Support System, Borgwaldt KC) to assess puffing behaviors.^12,42^ Carbon monoxide was collected (Vitalograph Inc) at each visit onset and before and after each cigarette.^12,34,35,42^ Subsequent sessions occurred every 5 ± 1 day, with a start time varying by 1 hour or less to control for diurnal variation. Procedures were identical to those used on day 1 except urine samples (biomarker measures) were collected only at the end of each period.

### Study Cigarettes

Participants purchased and smoked their preferred cigarette brand for 5 days to establish a baseline of smoking behaviors and exposure, serving as their own control.^12,42,43^ To briefly characterize the brands used during this period, 41% of the sample used Marlboro cigarettes, 13% Camel, 11% Maverick, 10% Pall Mall, 9% Newport, and 16% another brand; 62% used a full-flavored (ie, red) vs low machine-determined tar yield; and 51% used a 100-mm vs king-sized length rod. At day 5 before smoking the last cigarette of the session, participants received investigational SPECTRUM RNC cigarettes supplied free through the National Institute on Drug Abuse Drug Supply Program (coded by nicotine yield and tar yield, with NRC100 having the least nicotine and tar yield and NRC701 having the most). Participants received 20% more than their self-reported CPD (rounded to the nearest pack) to last between sessions, accounting for potential increased smoking or delays in attending sessions (rescheduling permitted ±1 day). Supplies were replenished at each visit. All participants received NRC400 5.2 mg/g cigarettes (RNC1) from days 5 through 20 and NRC200 1.3 mg/g cigarettes (RNC2) from days 20 through 35; order was not counterbalanced to maximize regulatory relevance for a nicotine reduction policy.^2,7^ Participants were blinded to the nicotine content change on day 20. Respective machine-determined cigarette nicotine yields were mean (SD) 0.26 (0.06) and 0.07 (0.02) mg; the tar yield of each was 9.0 (1.5) mg.

Verbal and print instructions explicitly stated that continued eligibility required smoking only study-supplied cigarettes, but that not all cigarettes needed to be consumed. At each visit, staff assessed non–study-supplied cigarette use and reconciled spent filters and unused cigarettes with those distributed at the previous session.^12,42^

### Measures

#### Nicotine Metabolism

The NMR was determined from blood samples^44^ based on previously identified quartile cut points^37,38,45,46^ associated with differences in smoking behaviors and toxicant exposure.^34^ We dichotomized the NMR as fast and slow using the upper (≥0.42, n = 59) and lower (≤0.26, n = 41) quartiles, respectively, to maximize sensitivity and exclude those quartiles between.

#### Analysis Covariates

Demographic and smoking history information assessed at the preliminary screening session included age, sex, race/ethnicity, body mass index, nicotine dependence (assessed using the Fagerström Test for Nicotine Dependence^47^), daily cigarette consumption over the past 7 days, and age when the participant started smoking regularly.

#### Primary Outcomes

##### Smoking Behaviors

Daily cigarette consumption and total puff volume (sum of all individual puff volumes per cigarette) were the primary smoking behavior measures; secondary puffing measures are included in Table 1. Daily cigarette consumption was assessed via self-report and verified through collection of spent filters, measures demonstrating high consistency in previous RNC^12^ and conventional^42^ cigarette studies (for this study, *r* = 0.96; *P* < .001; mean difference, 0.57; 95% CI, 0.50-0.65). Puffing behavior data were cleaned using standard procedures; exclusions eliminated 5% or less of the valid data for each variable.

**Table 1.** Outcome Measures by Cigarette Use Periods, Collapsed Across Nicotine Metabolism Groups

Outcome	Cigarette Use Period, Mean (95% CI)^a^		
	Preferred Brand	RNC1	RNC2
Smoking behaviors			
Daily cigarette consumption, No.^b^^,^^c^	13.9 (12.5-15.2)	16.5 (14.9-18.1)	14.8 (13.2-16.5)
Total puff volume, mL^b^^,^^c^^,^^d^	744 (681-806)	537 (479-595)	598 (547-649)
Puff count, No.^b^^,^^c^^,^^d^	14.7 (13.6-15.9)	11.2 (10.4-11.9)	12.3 (11.5-13.1)
Mean puff volume, mL	52.7 (48.1-57.4)	49.4 (44.4-54.3)	50.1 (45.5-54. 6)
Mean puff duration, s^c^^,^^d^	1.7 (1.6-1.8)	1.7 (1.5-1.8)	1.8 (1.7-2.0)
Interpuff interval, s^b^^,^^c^^,^^d^	26.1 (23.2-28.9)	22.3 (19.7-24.8)	20.7 (18.2-23.2)
Peak velocity, mL/s^b^^,^^c^^,^^d^	46.0 (42.7-49.4)	43.4 (39.8-46.9)	41.1 (37.9-44.4)
Biomarkers of exposure			
Onset CO level, ppm	19.1 (17.0-21.3)	19.4 (17.0-21.9)	19.7 (17.1-22.4)
CO boost, ppm^c^	4.4 (3.8-4.9)	4.6 (4.1-5.1)	4.2 (3.7-4.6)
NNAL, pg/mg creatinine^b^^,^^c^^,^^d^	280 (231-339)	229 (189-277)	190 (157-231)
TNE, nmoL/mg creatinine^b^^,^^c^^,^^d^	54.6 (48.1-62.1)	30.9 (26.0-36.6)	22.8 (17.8-29.0)

Abbreviations: CO, carbon monoxide; NNAL, 4-(methylnitrosamino)-1-(3-pyridyl)-1-butanol; RNC, reduced nicotine content of 5.2 mg (RNC1) and 1.3 mg (RNC2) of nicotine per gram of tobacco; TNE, total nicotine equivalent.

^a^ Data are presented as arithmetic mean (95% CI) except for NNAL and TNE, which are presented as geometric mean (95% CI).

^b^ Statistically significant difference between preferred brand and RNC1 cigarette periods at the *P* < .05 level.

^c^ Significant difference between RNC1 and RNC2 cigarette periods at the *P* < .05 level.

^d^ Significant difference between preferred brand and RNC2 cigarette periods at the *P* < .05 level.

##### Biomarkers of Exposure

To assess nicotine and nitrosamine exposure,^5,7,12,42^ urine samples were assayed for total nicotine equivalents (TNEs) (molar sum of nicotine; cotinine and 3′-hydroxycotinine and their glucuronides; and nicotine-N-oxide, cotinine-N-oxide, and nornicotine) and 4-(methylnitrosamino)-1-(3-pyridyl)-1-butanol (NNAL) (metabolite of the tobacco-specific nitrosamine 4-[methylnitrosamino]-1-[3-pyridyl]-1-butanone) using established liquid chromatography–mass spectrometry procedures^48,49^ and normalized for urine creatinine concentration. We used session-onset CO level to approximate general tobacco exposure and CO boost—the difference between precigarette and postcigarette CO assessments—to approximate exposure from a single cigarette.^12,42,50^

### Statistical Analysis

All variables were examined for normality; TNE and NNAL values were not normally distributed and were transformed using the natural logarithm. Unpaired *t* tests compared continuous sample characteristic and baseline outcome measures by the NMR and study completion status; χ^2^ independence tests compared categorical measures.

We created composite measures for repeatedly assessed outcomes by determining the mean of all assessments occurring within a study period. For example, RNC1 total puff volume was the mean of all total puff volumes during the period (day 5 second cigarette, both cigarettes on days 10 and 15, and day 20 first cigarette). We included only full days when calculating mean daily cigarette consumption (eg, RNC1 CPD = mean of days 6-19). Intraclass correlation coefficients examined the within-period consistency of these measures, and repeated measures analysis of variance examined within-period time outcomes. Intraclass correlation coefficients ranged from 0.77 to 0.97, indicating excellent agreement (intraclass correlation coefficients >0.75). Only baseline interpuff interval measures varied by time (*F*_1.78,146.20_, 5.26; *P* = .008): the second day 1 interpuff interval assessments were 2.9 and 2.5 seconds longer than the first interpuff interval on days 0 (*P* = .001) and 5 (*P* = .02).

Primary analyses were conducted using 6 linear mixed models (ie, per each main outcome) to account for correlated observations within individuals over time, using 2-tailed significance tests at the *P* < .0083 level to adjust for multiple outcomes. Separately, 5 exploratory models assessed the outcomes of secondary puffing behavior measures. Models included fixed-effect terms for study period (baseline vs RNC1 vs RNC2), NMR (slow vs fast), and their interaction. Given prior associations with RNC cigarette and NMR outcomes,^18,19,20,47,48,49,50,51,52,53^ covariates included sex, race (white vs nonwhite), body mass index, Fagerström Test for Nicotine Dependence total score (excluding CPD item for daily consumption analyses), and years smoking. All models used an unstructured covariance structure and Bonferroni-corrected pairwise comparisons for significant findings. A priori sample size was based on smoking behavior and biomarker differences between NMR group sizes of 25.^34^ Data analysis was performed from December 12, 2016, to January 3, 2018. Analyses were conducted using IBM SPSS Statistics, version 24 (SPSS Inc).

## Results

### Sample Characteristics

**Figure 2.**
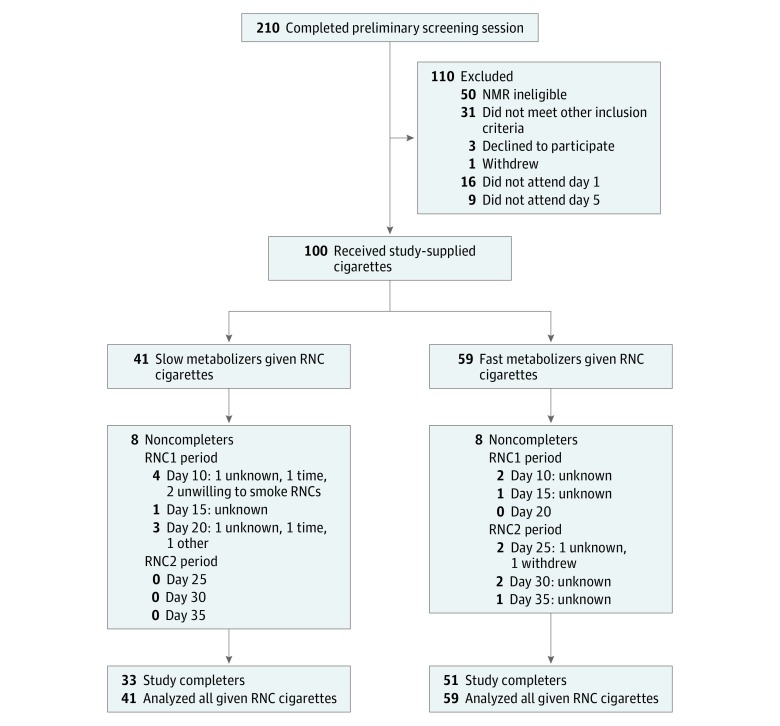
CONSORT Flow Diagram Depicting Study Recruitment and Retention NMR indicates nicotine-metabolite ratio; RNC, indicates reduced nicotine content of 5.2 mg (RNC1) and 1.3 mg (RNC2) of nicotine per gram of tobacco.

Of the 210 participants who attended the preliminary screening session, 109 met eligibility criteria and returned for day 1 (Figure 2). One hundred completed the 5-day, preferred-brand baseline period and received RNC cigarettes. Eighty-four completed the full study. Study completers and noncompleters did not differ significantly on any sample characteristics or by NMR.

Participants (73 [73.0%] men, 74 [74.0%] white), with mean (SD) age of 43.02 (12.13) years (range, 22-65 years), consumed 2.62 (95% CI, 1.54-3.70) more CPD during the RNC1 period vs their preferred brand during baseline (*P* < .001), reported smoking a mean (SD) of 17.31 (5.72) CPD (range, 10-40 CPD) and smoking regularly for a mean (SD) of 26.23 (12.11) years (range, 6-48 years), and were moderately nicotine dependent (mean [SD] Fagerström Test for Nicotine Dependence score, 5.48 [1.79]; range, 1-10). Fast metabolizers were more likely to be white (χ^2^_1_ = 18.74; *P* < .001), had greater baseline TNE (*t*_82_ = 2.02; *P* = .046) and NNAL (*t*_81_ = 2.46; *P* = .02) levels, had marginally lower body mass index (*t*_98_ = −1.82; *P* = .07), and were marginally less likely to be men (χ^2^_1_ = 3.47; *P* = .06) (Table 2). Other variables did not differ significantly by NMR.

**Table 2.** Sample Characteristics and Baseline Primary Outcome Measures by NMR Group

Characteristic	NMR Group, Mean (SD)^a^	
	Slow (n = 41)	Fast (n = 59)
Age, y	42.90 (12.39)	43.10 (12.04)
Sex, No. (%)^b^		
Men	34 (82.9)	39 (66.1)
Women	7 (17.1)	20 (33.9)
BMI^b^	28.47 (6.50)	26.33 (5.26)
Race, No. (%)^c^		
White	21 (51.2)	53 (89.8)
Black/African American	13 (31.7)	5 (8.5)
Other	7 (17.1)	1 (1.7)
Ethnicity, No. (%)		
Non-Hispanic	37 (94.9)	58 (98.3)
Hispanic/Latino	2 (5.1)	1 (1.7)
Cigarettes per day, No.	17.24 (6.98)	17.36 (4.72)
Years smoking	26.27 (12.47)	26.20 (11.97)
Nicotine dependence, FTND score	5.59 (1.75)	5.41 (1.83)
NMR^c^	0.19 (0.06)	0.57 (0.17)
Baseline outcome measures		
Cigarettes per day, No.	14.11 (5.32)	15.69 (6.07)
Total puff volume, mL	761.03 (255.00)	767.73 (298.08)
TNE, nmoL/mg creatinine^d^	56.26 (8.76)	75.19 (12.06)
NNAL, pg/mg creatinine^d^	217.02 (2.34)	343.78 (12.06)
Onset CO level, ppm	19.16 (9.72)	20.65 (8.21)
CO boost, ppm	4.03 (2.17)	4.32 (2.49)

Abbreviations: BMI, body mass index (calculated as weight in kilograms divided by height in meters squared); CO, carbon monoxide; FTND, Fagerström Test for Nicotine Dependence; NMR, nicotine-metabolite ratio; NNAL, 4-(methylnitrosamino)-1-(3-pyridyl)-1-butanol; TNE, total nicotine equivalents.

^a^ Data are presented as arithmetic mean (SD) for all continuous measures except NNAL and TNE, which are presented as geometric mean (SD); data are presented as number (valid percentage) for categorical measures.

^b^ Significant at the *P* < .10 level.

^c^ Significant at the *P* < .001 level.

^d^ Significant at the *P* < .05 level.

### Associations With Smoking Behaviors

#### Primary Measures

**Figure 3.**
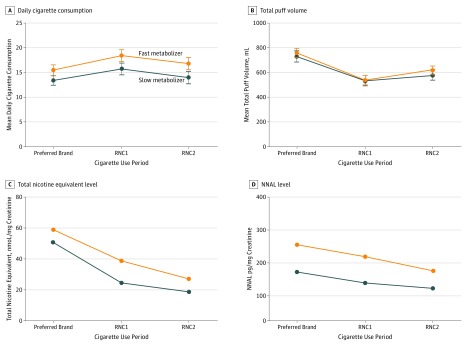
Differences in Primary Measures of Smoking Behavior and Urinary Biomarkers of Exposure Across Cigarette Use Periods by Nicotine-Metabolite Ratio Group Mean daily cigarette consumption (A) and mean total puff volume (B) depict arithmetic mean and SE. Total nicotine equivalent level (C) and 4-(methylnitrosamino)-1-(3-pyridyl)-1-butanol (NNAL) level (D) depict geometric mean only. RNC indicates reduced nicotine content of 5.2 mg (RNC1) and 1.3 mg (RNC2) of nicotine per gram of tobacco.

Both daily cigarette consumption (*F*_2,89.09_ = 36.33; *P* < .001) and total puff volume (*F*_2,99.56_ = 20.53; *P* < .001) differed significantly by study period (Table 1). Daily mean cigarette consumption during the RNC1 period (16.5 [95% CI, 14.9-18.1]) was greater than baseline (13.9 [95% CI, 12.5-15.2]) and the RNC2 period (14.8 [95% CI, 13.2-16.5]) (all *P* < .001); consumption during the RNC2 period was not significantly different from baseline (mean difference, 0.96 [95% CI, −0.36 to 2.28]; *P* = .24). Compared with baseline (mean, 744 mL [95% CI, 681-806 mL]), mean total puff volume decreased during both RNC periods (RNC1, 537 mL [95% CI, 479-595 mL]; RNC2, 598 mL [95% CI, 547-649 mL]; all *P* < .001). Total volume was greater during the RNC2 vs RNC1 period (*P* = .02). There was no main effect of NMR (CPD: *F*_1,106.81_ = 2.94; *P* = .09; total puff volume: *F*_1,97.67_ = 0.35; *P* = .56), nor did NMR moderate (CPD: *F*_2,89.09_ = 0.29, *P* = .75; total puff volume: *F*_2,99.56_ = 0.46; *P* = .64) associations with study period on these outcomes (Figure 3).

#### Secondary Measures

The NMR had no main effect and no interactive effect with study period on secondary puffing behavior measures. All secondary measures differed significantly by study period (ranges of *F*_2,87.72-96.89_ = 11.19-50.21; *P* < .001) (Table 1) except mean puff volume. Puff count decreased from baseline during both RNC periods but increased during the RNC2 period compared with the RNC1 period. Puff duration during the RNC1 period was not significantly different than baseline but increased during the RNC2 period compared with the RNC1 and baseline periods. Interpuff interval and maximum velocity decreased during the RNC1 and RNC2 periods compared with baseline; both outcomes decreased during the RNC2 vs RNC1 period.

### Associations With Biomarkers of Exposure

Urinary biomarkers differed significantly by NMR: fast metabolizers had greater overall NNAL (mean, 284 pg/mg creatinine; 95% CI, 226-361 pg/mg; *F*_1,83.26_ = 7.06; *P* = .009) and TNE (mean, 39.6 nmoL/mg creatinine; 95% CI, 32.5-47.9 nmoL/mg; *F*_1,91.42_ = 4.94; *P* = .03) than slow metabolizers (mean NNAL, 185 pg/mg creatinine; 95% CI, 144-240 pg/mg; mean TNE, 28.8 nmoL/mg creatinine; 95% CI, 22.9-35.9 nmoL/mg). The NNAL (*F*_2,82.70_ = 17.21) and TNE (*F*_2,83.21_ = 37.23) also differed significantly by study period (Table 1) such that both biomarkers decreased during both RNC periods (mean [95% CI]: RNC1 TNE, 30.9 nmoL/mg creatinine [95% CI, 26.0-36.6 nmoL/mg]; RNC1 NNAL, 229 pg/mg creatinine [95% CI, 189-277 pg/mg]; RNC2 TNE, 22.8 nmoL/mg creatinine [95% CI, 17.8-29.0 nmoL/mg]; RNC2 NNAL, 190 pg/mg creatinine [95% CI, 157-231 pg/mg]) compared with baseline (mean [95% CI]: TNE, 54.6 nmoL/mg creatinine [95% CI, 48.1-62.1 nmoL/mg]; NNAL, 280 pg/mg creatinine [95% CI, 231-339 pg/mg]; all *P* < .001) and during the RNC2 period compared with the RNC1 period (NNAL *P* = .001; TNE *P* = .003). The NMR × study period interaction was not significant for either outcome (TNE: *F*_2,83.21_ = 1.97; *P* = .15; NNAL: *F*_2,82.70_ = 0.70, *P* = .50) (Figure 3).

The NMR had no main or interactive effect with study period on CO measures. Carbon monoxide boost, but not onset CO, differed significantly by study period (*F*_2,101.88_ = 5.88; *P* = .004). Carbon monoxide boost was lower during the RNC2 (mean: 4.2 ppm [95% CI, 3.7-4.6 ppm]) vs RNC1 (mean: 4.6 ppm [95% CI, 4.1-5.1 ppm]) period (*P* = .003) although neither RNC period differed significantly from baseline (mean: 4.4 ppm [95% CI, 3.8-4.9 ppm]; RNC1 vs baseline *P* = .42; RNC2 vs baseline *P* = .90).

## Discussion

This study examined the associations of 2 RNC cigarette levels with multiple, repeatedly assessed measures of smoking behavior and biomarkers of exposure among slow and fast nicotine metabolizers. Both levels were associated with decreased puffing behaviors and urinary biomarkers but not cigarette consumption or CO level relative to smokers’ preferred brands. Contrary to our hypothesis, the NMR did not moderate these associations, suggesting that fast metabolizers are not at greater risk of increasing use or exposure with these products. The findings are consistent with those of prior RNC studies,^40,41^ although these smokers have greater conventional cigarette use and exposure.^34^ However, fast and slow metabolizers did not differ significantly in baseline smoking rate or dependence, as has been previously observed,^31,32,33,34,35,36,37,38,39^ but did differ significantly on biomarker exposure (ie, TNE, NNAL) as expected. It is possible that inclusion criteria requiring participants to smoke at least 5 CPD prevented NMR groups from differing in patterns of daily cigarette consumption. Nevertheless, results associated with the lack of a moderating association by the NMR have encouraging regulatory implications, as an RNC product standard may be implemented only if appropriate for the protection of public health and subgroups are not at increased risk of adverse consequences. Our findings suggest that a product standard similar to these nicotine levels is unlikely to negatively affect smokers with fast nicotine metabolism, as both groups responded to these products similarly.

Both RNC cigarette levels were associated with significant decreases in total puff volume, NNAL, and TNE relative to preferred-brand cigarettes. These results support the idea that 5.2-mg/g or lower RNC cigarettes significantly reduce puffing behavior and nicotine exposure among non–treatment-seeking adult daily smokers. These findings are consistent with studies^8,12^ using shorter cigarette use periods (ie, 7-10 days) and a 6-week multisite, randomized trial,^7^ suggesting that 7- to 15-day exposure periods are sufficient for understanding how longer-term RNC cigarettes affect these outcomes. The consistency of results across studies with varying RNC cigarette use periods is important for future research assessing the outcomes of other aspects of RNC cigarette use (eg, packaging, labeling) with limited exposure periods.

Neither RNC cigarette type was associated with significant reductions in daily cigarette consumption or CO levels compared with smokers’ preferred brands. Cigarette consumption findings replicate those of previous trials^5,7,8,9,12^ and may have been inflated by providing free cigarettes.^7^ Carbon monoxide results are consistent with some,^7^ but not all,^12^ studies. Discrepancies may reflect characteristics of the study-supplied cigarettes, such as their components or the absence of commercial branding (ie, packages lacked descriptors, and marketing claims were visually unappealing). Although CPD and CO levels were not reduced, it is important that the lowest RNC cigarette level did not increase these outcomes compared with baseline. Thus, when taken together with puffing behavior and biomarker results, the findings demonstrate no evidence of compensation at this nicotine content level.

Although these findings may appear to suggest that implementing a nicotine product standard at the levels tested will not produce meaningful reductions in smoking behavior or harm exposure at a population level, these results were obtained when providing non–treatment-seeking smokers with free cigarettes. These conditions do not fully mimic the environment of a national nicotine reduction policy, which would aim to reduce nicotine levels to an extent that compensation is not possible and produce population benefits through preventing experimental smokers from developing dependence and promoting cessation among current users. We did not expect, nor is it consistent with the larger RNC cigarette literature, to observe significant reductions in behavior from this intervention alone in this context. The results from this study simply support the evidence base demonstrating that very low levels of nicotine content do not increase smoking behaviors or harmful chemical exposures relative to preferred brand and thus have potential to reduce overall smoking prevalence if implemented alongside other effective tobacco control policies (eg, plain packaging, increased taxes, or ready access to noncombusted nicotine products).

### Limitations

This study has several limitations. First, the fast and slow NMR groups were not balanced by sex or race, although analyses controlled for these differences. Second, to thoroughly characterize outcomes of 2 RNC cigarette varieties, we did not test every available RNC level. Because the FDA has not specified the nicotine level of its intended product standard, this study adds to the evidence base used to understand 2 potential regulatory levels. Future studies should evaluate the lowest RNC cigarettes available (ie, 0.4 mg/g), which have the greatest efficacy in reducing smoking behaviors and exposure.^7^ Third, although RNC cigarette adherence was addressed at each visit, urinary biomarker values suggest that participants likely smoked non–study-supplied cigarettes, as we would expect to observe about a 48% and 87% reduction in TNE levels based on the reductions in nicotine content of the study cigarettes compared with conventional cigarettes. Instead, we observed respective TNE reductions of 46% and 58% from baseline during the RNC1 and RNC2 periods, suggesting that smokers may have more difficulty using cigarettes with markedly RNC when prohibited from using alternative sources of nicotine.

Although nonadherence may have attenuated associations with outcomes, this phenomenon is a common challenge in RNC cigarette trials.^7,54^ Because we did not use a traditional balanced crossover design with washout periods between conditions, we were unable to determine causal effects of RNC cigarettes on study outcomes; however, the current within-subject design requiring smokers to step down their nicotine content across multiple periods provides evidence with maximum relevance for a gradual approach to reduce cigarette nicotine content.

## Conclusions

This study contributes public health policy–relevant evidence suggesting that fast nicotine metabolizers are not at risk of increasing smoking behaviors or exposure when using 2 RNC cigarette levels. Thus, although neither RNC cigarette level tested had a significant association with reducing either daily cigarette consumption or CO levels, implementing an RNC product standard at these levels may decrease puffing behaviors and some tobacco exposure measures and may not negatively affect smokers with fast nicotine metabolism.

## Notes

Supplement. Trial Protocol
