# Supplementary material for: Association of Reduced Nicotine Content Cigarettes With Smoking Behaviors and Biomarkers of Exposure Among Slow and Fast Nicotine Metabolizers: A Nonrandomized Clinical Trial
Source: JAMA Netw Open. 2018 Aug 24;1(4):e181346. doi: 10.1001/jamanetworkopen.2018.1346 (PMC6322845; doi:10.1001/jamanetworkopen.2018.1346)
Supplement: Supplement. — Trial Protocol [file jamanetwopen-1-e181346-s001.pdf]

Application for Review of Human Research Section II IRB Protocol Summary [Version 1, 7/2/12]

***Principal Investigator*:** Andrew A. Strasser, Ph.D.

PROTOCOL TITLE
1. Full Title
Nicotine metabolism and low nicotine cigarettes

2. Brief Title
NMR low nicotine cigarettes

STUDY SPONSORSHIP
1. Funding Sponsor
National Institutes of Health, National Institute of Drug Abuse

PROTOCOL ABSTRACT
This study examines the effects of smoking low nicotine cigarettes on smoking behaviors and toxin exposure in fast and slow nicotine metabolizing smokers. We will recruit 100 current smokers (50 slow, 50 rapid nicotine metabolizing smokers) for a 35-day protocol. Participants must be 21 to 65 years old, smoking at least 10 cigarettes daily and have been smoking for at least 5 years. Participants will smoke their own brand cigarettes during a baseline 5 day period, followed by a 15-day period of smoking low nicotine content cigarette level 1: 0.25 mg nicotine content, followed by a 15-day period of smoking low nicotine content cigarette level 2: 0.08 mg nicotine content.

OBJECTIVES
1. Overall Objectives
This application is designed to provide empirical science to inform the FDA on the effect smoking low nicotine content cigarettes will have on use patterns and harm exposure. The study is designed to ask two important questions:
1) Will individuals smoke LNC cigarettes more intensely or smoke more each day, thereby maintaining their desired nicotine levels, and as a result continue to be exposed to significant toxin levels?
2) Are there subgroups of smokers more vulnerable to these adaptive smoking behaviors with LNCs and resulting associated risks?

2. Primary Outcome Variable(s)
1) Smoking topography (total puff volume)
2) Daily cigarette consumption
3) Toxin exposures (NNK, mercapturic acid metabolites, total nicotine equivalents)

2. Secondary Outcome Variable(s)
1. Carbon monoxide measurements taken before and after cigarettes smoked at each visit

2. To identify sub-groups of the smoking population who may exhibit greater compensatory smoking and/or carcinogen exposure

BACKGROUND
1. Family Smoking Prevention and Control Act
The Family Smoking Prevention and Control Act of 2009 gave the United States Food and Drug Administration (FDA) authority to regulate tobacco products, including cigarettes, roll your own tobacco and smokeless tobacco products. FDA's regulatory authority encompasses defining standards for nicotine yields and the reduction or elimination of other harmful substances present in tobacco products, when deemed to be in the interest of the public's health (see Sec. 907, Tobacco Product Standards). While many studies are underway to determine the effects of varying nicotine yields on smoking behavior and toxins,

studies performed at a population level may not identify significant harms that exist in subgroups of smokers. The proposed research will fill this gap by determining how risk varies in smokers with slow versus fast nicotine metabolism, a heritable trait known to alter smoking behaviors and resulting toxin exposure.

2. New Low Nicotine Content (LNC) Cigarettes

The latest regulatory efforts by the FDA include trying to identify nicotine content levels in cigarettes that will reduce addiction and improve the public's health. This goal has led to production of research cigarettes with varying nicotine and tar content (new low nicotine content, LNC). We propose in this application to apply our research expertise of traditional low nicotine yield (light) cigarettes [1, 14], and Quest LNC cigarettes [2, 3], to examine smoking behaviors and toxin exposures when smoking these new low nicotine content cigarettes. An innovative and important aspect of this proposal is a focus on understanding how heritable individual differences in the rate of nicotine metabolism can influence the use of these, and resulting harmful exposures, of this new product.

3. The role of variable nicotine metabolism

Nicotine is converted to cotinine, and cotinine is converted to 3'hydroxycotinine, and both of these processes are influenced primarily by the CYP2A6 enzyme [20-22]. Our collaboration on this project, Dr. Tyndale's laboratory (UToronto), has characterized a number of null and reduced activity *CYP2A6* gene alleles that alter nicotine metabolism rate in vivo [23, 24] as well as ethnic variability in *CYP2A6* allele frequencies [25-27]. Faster metabolizers of nicotine, those with two fully functional alleles, smoke more cigarettes per day [28], puff more vigorously on their cigarettes [6], are more dependent [29, 30], and have higher rates of lung cancer [30]. However, given that CYP2A6 alleles account for only part of the inherited variability in nicotine metabolism [22]) and that there are environmental influences on nicotine metabolism [31], we utilize a genetically informed biomarker of nicotine metabolism, which accounts for genetic and environmental influences on nicotine metabolism [11].

Nicotine metabolism ratio (NMR) is the simple ratio of 3-hydroxycotinine/cotinine from smokers, is noninvasive, independent of time since last cigarette, stable and reliable [23, 32]. Based on NMR, we have previously shown fast metabolizers are significantly less likely to quit smoking on placebo [10], and nicotine patch [8, 9] but are successful at quitting with non-nicotine medications [10]. Based on these independent clinical trials, we have determined the slow metabolizers to have an NMR ≤ 0.26 (approximately 25% of the smoking population), while normal metabolizers have higher NMRs. For this application to maximize sensitivity we will compare slow metabolizers (NMR ≤ 0.26) to rapid metabolizers (NMR $\geq .42$).

4. Smoking Topography and Toxin Exposure

The UPENN Center for Interdisciplinary Research on Nicotine Addiction (CIRNA) has conducted extensive work on examining the relationship between smoking behaviors and toxin exposures highlighted by our publications which report an association between total puff volume and NNAL [5], total puff volume and CO boost [2, 14], puff velocity and CO [1] and puff volume and tar exposure [3]. Results from this line of published studies, which represent over 650 topography and toxin sessions, demonstrate the need to meticulously assess smoking behaviors when examining exposures from cigarette smoking, and we propose to extend this research question herein.

5. Toxin Exposures

Differences in composition and burning characteristics of cigarettes and differences in smoking behavior (intensity of smoking) can affect the pattern and levels of exposure to various tobacco smoke toxins. Biomarker measurement is the optimal way to assess exposure to tobacco smoke toxins. It is desirable to measure a breadth of toxins, as is proposed in this study. Vapor phase biomarkers of interest include carbon monoxide and a number of volatile organic compounds. Carbon monoxide can be measured in expired air [14, 47]. Volatile organic chemicals, including benzene, acrolein, acrylamide, 1,3 butadiene, ethylene oxide, propylene oxide and crotonaldelhyde, can be measure in the urine as mercapturic acid metabolites [48, 49]. These compounds represent important carcinogens and cardiovascular toxins in tobacco smoke. Particulate measures include tobacco specific nitrosamines (NNK; measured as urine

NNAL) and polycyclic aromatic hydrocarbons, which represent other carcinogens in smoke. Nicotine intake is of course important to measure with respect to addiction potential. Nicotine intake can be estimated using the sum of nicotine metabolites in urine; and for within subject studies, by cotinine measurement. Dr. Benowitz' laboratory (UCSF, Co-investigator) has been one of the key contributors to conducting assays to quantify toxicant and nicotine exposures, and will oversee the work proposed in this application as an extension of the ongoing work of the parent grant.

CHARACTERISTICS OF THE STUDY POPULATION

1. Target Population

Adults who smoke at least 10 cigarettes daily, have been smoking for at least 5 years and who are not currently trying to quit smoking, and who are characterized as a slow nicotine metabolizer (NMR ≤ .26) or rapid nicotine metabolizer (NMR ≥ .42), as determined by results from a blood test at the initial session.

2. Accrual

Recruitment will take place via several sources: community and campus bulletin boards, local newspapers, and previous participants who have agreed to be contacted for non-cessation and/or non-treatment studies. Dr. Strasser's research team has recruited 210 smokers in a 35-day, 8 session, 2-hours/session protocol that closely matches the proposed project, in the past 5 years.

<u>Justification of Sample Size:</u> Preliminary data [2]; (R01 120594) of 250 smokers suggests total puff volume to be approximately 563 ml (SD=136) and 660.0 ml (SD=145) for own versus low nicotine cigarettes respectively; and cigarettes per day at 18 vs. 22 (SD=5); and NNAL levels of 1.18 pmol/ml and 1.61 pmol/ml (SD=.44), for the within-subject comparison. The between subject (NMR) comparison is based on slow vs. rapid NMR [683 ml vs. 874 ml (SD=250)] from Strasser et al., 2011 [5]. Urinary biomarkers are collected at D5, 20, 35. Due to the long half-life of the biomarkers, changes do not occur until after 5-8 days (half-lives vary) and will only be collected at the end of each period, the beginning of Day 5 (own brand), Day 20 (end of LNC1, or end of little cigar), and Day 35 (end of LNC2). Cotinine and NNAL are highly correlated within subject (0.5); [83] and represent a modest effect size relative to the proposed biomarker panel. Therefore we use NNAL observations from previous work in our lab, to calculate power and sample size. Powering for the interaction test (rapid nicotine metabolizers will exhibit greater compensatory smoking and greater toxin exposure compared to slow metabolizers), and correcting alpha for multiple comparisons with 80% power, 50 participants per group will sufficiently detect significant differences for all behavioral and biomarker outcomes (NCSS:Power-Sample Size).

4. Key Inclusion Criteria

1) adults age 21-65.
2) smoking at least 10 cigarettes per day.
3) smoking daily for the last 5 years.
4) Provide a baseline breath CO reading equal to or greater than 10 parts per million.
5) smoke predominantly non-menthol filtered cigarettes (research cigarettes are only non-menthol).
6) not currently using any other nicotine containing products such as cigars, smokeless tobacco, nicotine replacement therapies (patch or gum).
7) are fluent in English and are capable of providing written informed consent, which includes compliance with the requirements and restrictions listed in the combined consent and HIPAA form.

Participants who meet the inclusion/exclusion criteria and attend an Intake Visit for medical screening will be asked to provide a blood sample to determine NMR. Only those individuals who are characterized as slow or rapid, based on NMRs ≤ .26, or ≥ .42, will be eligible.

5. Key Exclusion Criteria

1) use of any nicotine containing products other than cigarettes
2) current or impending enrollment in smoking cessation program

3) history in the last year or current treatment of substance abuse (other than nicotine dependence)
4) positive drug screen for cocaine, methamphetamines or opiates for urine drug screen at intake session
5) alcohol use greater than 25 standard drinks per week
6) current or planned pregnancy or lactating
7) history or current diagnosis of psychosis, bipolar disorder, mania, or schizophrenia
8) current major depression (history of major depression but in remission for a minimum 6 months is eligible).
9) serious or unstable disease within past year (e.g. cancer other than melanoma, heart disease)
10) history or current diagnosis of COPD
11) history of stroke or heart attack
12) age less than 21 years- We wish to recruit smokers with well established smoking patterns and will restrict recruitment to those over 21
13) age more than 65 years - smoking is often associated with cardiovascular and pulmonary obstructive diseases that manifest later in life. As smokers develop these problems, smoking behavior and biomarkers of harm may be affected therefore we will restrict enrollment to those under age 65
14) provide baseline CO reading less than 10 at initial session
15) current use (or use within past 14 days) of any medication that affects the biotransformation of nicotine, such as anticonvulsant drugs, rifampin, and disulfiram; or any psychoactive medications which can affect smoking behaviors
16) any conditions viewed by the PI or study physician as creating a potential increased risk to a participant due to their participation will be reasons for exclusion
17) at the discretion of the PI, any participants viewed as non-compliant will be excluded from further participation

6. Vulnerable Populations

Children, pregnant women, fetuses, neonates, or prisoners are not included in this research study.

7. Populations vulnerable to undue influence or coercion

Educationally or economically disadvantaged persons or cognitively impaired persons are not intentionally recruited in the current study. Because recruitment efforts for this study will be targeted to the greater Philadelphia area, University of Pennsylvania employees and students may view these advertisements and choose to respond. Status of participation in the current study will be independent of the participants' work or school activities.

8. Subject Recruitment

Recruitment will be through two methods: previous center participants who agreed to future contact will be contacted to assess interest in participation and prospective participants will be recruited through advertisements in local media outlets (radio, TV, newspaper, internet).

STUDY DESIGN

1. Phase

Not applicable.

2. Design

A 2x3 design with a between-subject factor of NMR (slow NMR 0.26 vs. rapid NMR .42) determined prior to enrollment and a within-subject factor of own brand of cigarette vs. LNC 1 (0.25 mg nicotine content, 9.0 mg tar), and LNC 2 (0.08 mg nicotine content, 9.0 mg tar), designed to test of the effects of cigarette nicotine level on smoking behaviors and toxin biomarkers. Additionally, the 5-day ad libitum observational period permits the participant to importantly be characterized for reliability and daily patterns of smoking behaviors, as well as allowing them to become accustomed to the smoking topography device (4

cigarettes in 2 lab visits) and other procedures, as well as provide a baseline (control) for biomarker level (Day 5). Therefore, the proposed design is optimal in that it allows us to assess usual smoking (Days 1-5), as well as directly compare the effects of interest thus maximizing statistical power. Study visits are described as Intake Visit, Sessions 1-8 where Session 1 is Day 1, S2 is Day 5, S3 is Day 10 and so on through Session 8, Day 35.

3. Study Duration

Timeline

To enroll subjects and complete the study, estimated 1 year. Subject participates in a study for initial screening phone call, then initial visit before final eligibility is determined. After eligibility is confirmed, study visits will be scheduled for an additional 5 weeks (8 visits total) starting with the next visit. Total participation time is approximately 2 months. Study is proposed for September 2012 start.

Drugs or Devices

Study Cigarettes will be provided by NIDA as part of their research cigarette production program.

STUDY PROCEDURES

1. Procedures

**TABLE 1** MEASURES	**STUDY DAY**								
	Intake	1	5	10	15	20	25	30	35
**SMOKING BEHAVIORS**									
SMOKING TOPOGRAPHY		X	X	X	X	X	X	X	X
USED CIG. FILTERS COUNT			X	X	X	X	X	X	X
DAILYCIG CALENDAR REVIEWED		X	X	X	X	X	X	X	X
NICOTINE METABOLISM RATIO	X								
**BIOMARKERS**									
CARBON MONOXIDE		X	X	X	X	X	X	X	X
TOTAL NICOTINE EQUIVALENTS			X			X			X
TOXIN BIOMARKERS			X			X			X
**SUBJECTIVE MEASURES**									
DEMOGRAPHICS + PREFERRED CIG BRAND + SMOKING Hx.	X								
NICOTINE DEPENDENCE	X								
CIG EVAL (CES, VAS) + SENSORY (SQ) WITHDRAWAL (WSC) + QSU		X	X	X	X	X	X	X	X

Initial eligibility will be determined during a telephone interview by an experienced research technician. For participants who meet eligibility, an Intake session will be scheduled.

Participants must bring in a pack of their own cigarettes to the intake session to verify their cigarette brand type. At the intake session, participants will arrive at the laboratory, sign informed consent, complete baseline, descriptive and smoking history questionnaires (Table 1), provide a blood pressure reading, breath alcohol (BAC) and carbon monoxide (CO) samples and have a blood sample drawn to determine NMR. Blood samples will be shipped to Dr. Tyndale's lab (U. Toronto). Participants will also provide a urine drug screen (at least 30mL [two tablespoons] of urine) and urine pregnancy test (female participants of childbearing potential only) will be administered. The urine drug screen will assess the recent use of cocaine, methamphetamines and/or opiates. Participants that test positive for cocaine, methamphetamines, or pregnancy will be ineligible. If a participant is taking a prescribed opiate for pain management for a specific, time-limited purpose (e.g., dental surgery) the study physician may determine that the participant is in fact eligible to continue in the study. Participants will be informed that their final eligibility will be determined by the NMR results from their blood sample and that they will be told of their eligibility by telephone. Duration 2 hours.

If eligible, participants will be scheduled to a study track of 8 sessions scheduled to start at the same time of day to reduce diurnal variability. Ideally all sessions will start within an hour of the same time, however if participants are unable to attend a session as scheduled, sessions will take place as soon as possible to maintain the schedule. Sessions will be spaced as close to 5 days apart as possible, with up to 2 days variation to account for participant scheduling.

On Day 1, participants will provide breath BAC and CO samples, complete measures and receive instructions on collecting and storing used cigarette filters in date- and time-labeled bags.Participants will smoke a baseline cigarette at the beginning of each session use the smoking topography device in the lab to smoke two cigarettes interspersed by 30 minutes. After the session, participants will collect all spent cigarette filters until the Day 5 laboratory visit. Participants supply their own cigarettes for the 5-day observational period. Duration: 2 hours.

On Day 5, participants will attend a laboratory session where they will return used cigarette filters in date and time marked bags, smoke two cigarettes using the smoking topography device, interspersed by 30 minutes, provide BAC, CO, urine samples in addition to completing cigarette ratings questionnaire. Participants will be provided with their LNC1 supply and date- and time-labeled bags to collect filters. Duration: 2 hours.

Days 10 and 15. Participants will complete their smoking topography session, have their cigarette usage reviewed, and be supplied with their research cigarettes. Participants will complete BAC and CO samples, subjective measures and confirm their next appointment. Duration: 2 hours.

Day 20 procedures will be identical to Day 5, with the exception that participants will be switched to the LNC2 level. Urine and CO samples will be collected as on Day 5. Duration: 2 hours.

Days 25 and 30 procedures will be procedurally identical to Day 10.

Day 35 procedures will be procedurally identical to Day 5 with BAC, CO and urine samples collected. participants will be debriefed on study purpose. Duration: 2 hours.

Description of Measures and Variables

<u>Predictor and Covariate Measures</u>

1. Demographic and Smoking History Measures. Age, sex, race, height and weight, years smoking, and cigarettes per day will be collected at baseline. The Fagerström Test for Nicotine Dependence (FTND) [77] will be used to measure nicotine dependence, and is a 6-item measure with satisfactory internal consistency (Cronbach's alpha=.64) and high test-retest reliability (r= .88) [78]. Smoking urges

(QSU); [79] and Withdrawal [80] will be assessed at each session, used as a covariate, and in exploratory mediator analysis.

2. Nicotine Metabolism Ratio. NMR will be determined from the 3-hydroxycotinine and cotinine results of the blood sample, and used to characterize participants as slow NMR (≤ 0.26), or rapid NMR (≥.42).

3. Cigarette Characteristics. Participants will provide information on their own cigarette brand by completing a cigarette characteristics questionnaire at baseline, including: cigarette size (king, regular, 100, 120, other), filtered/non-filtered, soft/hard pack, tar-nicotine level, and menthol/non- menthol, cigarette name brand.

4. Withdrawal Symptoms (WSC-W). A Withdrawal Symptom Checklist will be used to measure withdrawal symptoms associated with quitting smoking (Hughes et al., 1984). The checklist consists of 18 items such as cravings, irritability, difficulty concentrating, restlessness, impatience, anxious/tense, insomnia, drowsiness, nausea, tremors, increased heart rate, general physical complaints (e.g., sweating, dizziness), increased hunger, increased eating, headache, gastrointestinal disturbance, depression, and fatigue. Participants will rate the intensity of their symptoms on the following scale: 0 = not present, 1 = mild, 2 = moderate, 3 = severe. This will enable us to test whether differences in cigarette nicotine level affect tobacco withdrawal symptoms, and whether such symptoms relate to compensation. The WSC-W will be administered at all visits except the Intake.

5. Questionnaire on Smoking Urges (QSU). The QSU, a 32-item Likert-format self-report instrument will be used to assess smoking urges and craving (Tiffany and Drobes, 1991). Questionnaire items are separated into four areas related to smoking urge: 1) desire to smoke, 2) anticipation of immediate positive outcome from smoking, 3) anticipation of immediate relief from nicotine withdrawal or relief from negative affect, and 4) intentions to smoke. Participants will be asked to report how strongly they agree or disagree with each of the 32 statements (e.g., “smoking would make me feel very good right now”). Scoring uses a two-factor solution; factor I items primarily reflect intention and desire to smoke and the anticipation of positive reinforcement, while factor II items reflect anticipation of negative reinforcement.

Outcomes: Behavioral and Biochemical Measures

Cigarettes will be smoked using the Borgwaldt KC (Richmond, VA; formerly Clinical Research Support Systems) portable smoking topography machine. This device is specifically designed to collect smoking topography variables. A cigarette is placed in the sterilized flowmeter mouthpiece, and the internal pressure transducer measures pressure changes that occur during inhalation. The pressure changes are amplified, digitized and sampled at 1000 Hz, then software converts the signal to airflow (ml/s) in real time (s), and from this provides number of puffs, puff volume, puff duration, maximum flow, and interpuff interval (time between puffs). The device is about the dimensions of a US cigarette pack, is lightweight, and offers the advantage of data collection in the smoking environment.

Urine Sample. At the beginning of Days 5, 20 and 35 participants will be provided with a 4-ounce (118-mL) polyethylene specimen collection jar (Fisher Scientific, Pittsburgh, PA). Each sample will be given a unique label, and maintained at -20 °C until analysis at Dr. Benowitz' laboratory. Samples will be assayed only on participants who complete the 35-day protocol. Samples will be aliquot for analyses (toxins and TNEs) and reserves, which will be stored and later examined for exploratory biomarkers based on publication of new research.

CO measures will be assessed at the onset of each visit. CO measures will be made using a Vitalograph Breath CO Analyzer (McNeil International., Lenexa, KS). A new, disposable mouthpiece will be provided for each participant. The device has a digital screen which reports CO in parts per million (ppm). Participants will provide a CO breath sample using standard procedures [1, 14]; the largest

reading will be recorded as the CO level.

Subjective Measures

Visual Analog Scale (VAS). To measure the subjective effects of the subject's preferred brand, their initial exposure to each low nicotine cigarette, we will use the VAS measure, a 100 mm analog scale that has been used in previous smoking studies (1,2). Items include: strength, heat, draw, smoke smell, and burn rate. Composite ratings will be calculated in identical fashion to those described for the smoking topography outcome measures.

Cigarette Consumption and Adherence Measures

At each visit after Day 5, participants will be provided with 20% more cigarettes than their reported baseline daily consumption in order to permit compensatory smoking by increasing daily cigarette consumption. Also, participants will be given a resealable plastic bag for each day of the study until their next laboratory visit. They will be asked to place every spent cigarette butt from each day into a labeled bag and return the bags at their next visit. A research assistant will count the cigarette butts and compare it to the participant self-reported number of cigarettes per day smoked. By collecting used cigarette butts we hope to better assess the actual number of cigarettes smoked per day. This measure is important because participants may compensate for less nicotine by smoking more daily cigarettes. Total number of cigarettes smoked and mean number of daily cigarettes smoked will be determined for each cigarette nicotine level. Participants will be asked at each visit if they have smoked any cigarettes other than the ones provided to them by the study staff, and if so, how many. Evidence of compensatory smoking at the cigarette level will be determined by differences from baseline. Participants will be asked at the conclusion of the study if they had used any cigarettes other than the ones provided to them by the study staff.

Exploratory Analyses

To compare against the NMR result, we propose to perform genotyping for polymorphisms in genes involved in the metabolism and elimination of nicotine (e.g., *CYP2A6, CYP2B6, UGTs*) and those potentially involved in the pharmacodynamic effects of nicotine (e.g., nicotinic receptors and dopamine pathway genes). We may genotype for additional polymorphisms in genes that may become identified as associated with nicotine dependence and related phenotypes in ongoing research.

2. Statistical Analysis

Our primary behavioral outcome measures are total puff volume and daily cigarette count. Our biomarker outcome measures are the toxin exposures and total nicotine equivalents collected at Days 5, 20, and 35. These measures are continuous, and previously been observed to meet assumptions of normality; except cigarette count, which will be treated as a negative binomial count. Analysis will be conducted in a mixed-effects regression framework, using the procedure xtmixed in Stata (Stata Corp., TX), or procedure xtnbreg for cigarette counts. This approach has been previously used in our projects on smoking behaviors and toxin exposure, and is ideally suited to test our hypotheses because this approach controls within-subject variability by modeling explicitly the correlation of observations within subject. Also, the mixed-models framework permits the flexibility to add covariates, and to allow for heterogeneity of error variances among different sessions or different subgroups, e.g., NMR groups

3. Data Storage and Confidentiality

- **Paper-based records will be kept in a secure location and only be accessible to personnel involved in the study.**
- **Computer-based files will only be made available to personnel involved in the study through the use of access privileges and passwords.**
- **Prior to access to any study-related information, personnel will be required to sign statements agreeing to protect the security and confidentiality of identifiable information.**
- **Wherever feasible, identifiers will be removed from study-related information.**

Communications made among study staff regarding participants will use identification number only and

not include names or any other personal identification numbers. Participant data will be kept in locked files which are only accessible to staff. In all data sets we will only include identification numbers to label each participant. No names or other unique identifiers will appear in data sets. Only study-specific staff, identified in institutional review board-approved materials, will have access to the list of names matched to identification numbers.

Subject Privacy

Study materials will use ID numbers to identify participants. All paper data is kept in locked files. Phone and email communication between staff will use ID number only to identify participants. All data sets use ID numbers only. Individuals will interact with study staff over the phone, by email, and in person in the lab. Staff at the University of Pennsylvania who may need to have access to the participants' name, and other information in the course of their duties (research oversight, compensation/business office) may have access to study information. 1) study materials will not include any information that identifies individuals participation, and any e-mail communication uses ID numbers and never names or personal information; 2) results are never communicated to study staff or participants; 3) all paper data is kept in locked files; 4) all data sets use ID numbers only; 5) study samples will be labeled with coded ID numbers.

Data Disclosure

Will the data be disclosed to anyone who is not listed under Personnel?

The Food and Drug Administration, The Office of Human Research Protections, The National Institutes of Health may request reviewing data from this study. If participants agree, a subset of their deidentified data may be deposited into the Pharmacogenetics Research Network Database (PharmGKB) or another appropriate database on the web.

Personal Health Information to be collected includes:

- Name, address, telephone number, date of birth
- Email address
- Social Security number (for compensation purposes)
- Personal medical history
- Results from research tests and procedures
- Information on tobacco related biomarkers
- Genetic information from blood samples

3. Tissue Specimens

Urine. A urine sample will be required at the Intake Session for drug and pregnancy screenings. Participants who test positive for study prohibited drug use will be deemed ineligible, as will women who have a positive pregnancy test. Urine samples from Days 5, 20, and 35 will be used to assess biomarker levels of cigarette smoke constituents. Urine Samples will be transferred to Dr. Benowitz' lab at the University of California at San Francisco for analysis. Samples will be number coded prior to shipment; therefore there will be no way for the UCSF lab to identify individual study participants.

Blood. Because, on average, the quality of DNA that is extracted from blood is superior to the quality of DNA that is extracted from saliva, we are asking participants to provide 3 tubes of blood (up to 30ml or 3 tablespoons) at the Intake Session for genetic analysis. Given that the complete predictive validity of the genotypes under study are not fully known at this time, participants will not be informed of the test results. All specimens are to be collected solely for research purposes. Blood samples will be transferred to Dr. Tyndale's laboratory at the University of Toronto for analysis. Samples will be number coded prior to shipment; therefore there will be no way for the University of Toronto lab to identify individual study participants.

Our research team considers data sharing to be a major priority. Therefore, samples for genetic analyses will be stored for future use. At the Intake Session, blood for the NMR will be collected in conjunction with smoking phenotypes, and subject demographics. While we do not propose to study smoking phenotypes (i.e., dependence) extensively as a primary goal in this trial, we are aware that this represents a large sample set in which to do this, collected in a standardized format. This will be an extraordinary resource for genome wide association studies (GWAS) and next-generation sequencing studies. This resource will be useful for the addiction research community as well as for other Pharmacogenetics Research Network and research communities where having a large group of healthy smokers provides much needed control groups (e.g., lung cancer, schizophrenia).

Our study is in compliance with NIH's data sharing mandate. Specifically, we will follow the guidelines provided for the NIH Genome Wide Association Studies and Next Generation Sequencing, and related programs. The data sharing policy includes the following features:

- All human protocols and consents will include IRB-approved language for data sharing.
- Study related documents and de-identified subject data may be shared upon request and reviewed by the study investigators.
- Raw study data and biospecimens for all projects will be available to the scientific community within 2 years of study completion (or sooner), for collaborative research with investigators on the parent grant, Pharmacogenetics of Nicotine Addiction Treatment (PNAT), Penn IRB# 811772. Qualified investigators who wish to access this material will complete a form available on the PNAT website, which includes the description of the proposed study and delineates specific uses. The review and approval of data use is essential to ensure that samples are tested only for assays and phenotypes that are consistent with original consent forms. These requests will be reviewed and approved by the PNAT Data Sharing Committee.

4. Genetic Testing

This study involves testing blood samples to identify NMR for participants and examination of DNA from blood samples to evaluate additional genetic variation related to smoking behavior and treatment outcomes. All biospecimens will be numerically coded and any information linking this numeric code to the participant's protected health information will be kept separate and secure as described above. No information concerning the results of this testing will be shared with the study participants.

RISK/BENEFIT ASSESSMENT

1. Potential Study Risks

Confidentiality and Loss of Privacy. Communications made among study staff regarding participants will use identification number only and not include names or any other personal identification numbers. Participant data will be kept in locked files which are only accessible to staff. In all data sets we will only include identification numbers to label each participant. No names or other unique identifiers will appear in data sets. Only study-specific staff, identified in institutional review board-approved materials, will have access to the list of names matched to identification numbers.

Assessments. Some subjects may experience some emotional distress during the assessments due to learning their carbon monoxide levels, seeing how many cigarettes they smoke. These events happen very rarely and in almost all cases are short-lived and of low intensity.

Cigarette Smoking. Although cigarette smoking is associated with many diseases, we do not believe the risk is beyond every day risk as all participants must be smoking 10 daily cigarettes to be eligible. In addition, we will minimize any risks related to the loss of privacy by maintaining strict confidentiality.

Blood Draw. We use trained phlebotomists to collect blood samples from participants. In some occasions, bruising, slight bleeding, and discomfort may occur at the needle site. In rare instances, participants may feel faint. These events are typically short lived and the participant quickly recovers.

2. Potential Study Benefits

There are no direct benefits to subjects as a result of participating in the study. Cigarette smoking remains the greatest preventable cause of disease and death in the United States. Although many smokers try to quit each year, the vast majority fail. The proposed study is a novel investigation to better understand how low nicotine content cigarettes and little cigars affect exposures, particularly in sub- groups that may be at greater risk of exposure based on inherited individual differences. Results may have clinical implications to demonstrate the relative increase in exposure in sub groups of smokers; and the proposed project has massive potential public health benefits by being able to provide empirical support for FDA policy on nicotine regulation in tobacco products.

3. Alternatives to Participation

The alternative to participation is to decide not to enroll in the study.

4. Data and Safety Monitoring Plan

The Principal Investigator, Dr. Andrew A. Strasser, Ph.D., will be responsible for overseeing and completing the monitoring process. During the course of the study, safety and data quality monitoring will be performed on an ongoing basis by the Research Coordinator and Principal Investigator. The Research Coordinator will be responsible for collecting and recording all data. The Principal Investigator will be responsible for ensuring all source documentation exists for the data in the case report forms and that all corrections are done according to the principles of Good Clinical Practice.

The Principal Investigator will be responsible for assuring that all staff and participants understand and accept the obligations incurred in undertaking this human behavioral pharmacology study in accordance with 21 CFR Parts 312, 511, 812, 813 and any other applicable regulations; the obligation to obtain informed consent in accordance with 21 CFR Part 50; the obligation to obtain IRB review and approval of a clinical investigation before the investigation may be initiated and to ensure continuing review of the study by the IRB in accordance with 21 CFR Part 56.

Monitoring for adverse events will be conducted in real time by Dr. Strasser. The only drug being administered is the nicotine from the cigarettes. While the cigarettes contain toxic and carcinogenic materials, the health risks of smoking these cigarettes should not exceed typical smoking of the study participants who must report smoking a minimum 10 cigarettes daily to be eligible.

Any adverse event case will be reviewed by Drs. Strasser and Leone. After removal of identification information, all serious adverse events will be reported to the University of Pennsylvania Institutional Review Board and the funding agency within 24 hours. All adverse events will be recorded and a summary table reviewed by the IRB annually. All research personnel associated with this study have completed the University of Pennsylvania's Patient Oriented Research Training Program, or their respective university equivalent, or the NIH patient oriented research training program, as well as HIPAA Compliance Training**.**

5. Risk/Benefit Assessment

This study poses minimal risks to subjects participating, however it has the potential to benefit society by measuring the effects of changing nicotine levels in cigarettes. Results may have clinical implications to demonstrate the relative increase in exposure in sub groups of smokers; and the proposed project has massive potential public health benefits by being able to provide empirical support for FDA policy on nicotine regulation in tobacco products.

SUBJECT COMPENSATION

Subjects will be compensated $590 total for their participation, including returning all cigarette butts and urine samples, and completing all laboratory sessions. Compensation will be paid in cash at each in-person visit according to the table below.

Day	Completing Visit	Transportation	Task completion*	Total

Intake	10	10		20
1	20	10	10	40
5	30	10	20	60
10	40	10	20	70
15	40	10	20	70
20	60	10	20	90
25	40	10	20	70
30	40	10	20	70
35	70	10	20	100
				590

INFORMED CONSENT

1. Consent Process

Informed consent will be obtained in person at the beginning of the intake session in a private lab room by a trained research assistant. After verifying contact information, the research assistant will read the consent form to the participant and answer any participant questions. If the participant has difficulty or is unable to understand the consent form or other procedures, they will be excluded from the study.

2. Waiver of Authorization

N/A

RESOURCES NECESSARY FOR HUMAN RESEARCH PROTECTION

Qualifications of Investigators. Brief highlights are presented below for key investigators.

Andrew A. Strasser, Ph.D., Site-Principal Investigator, Dr. Andrew A. Strasser, Ph.D. is an Associate Professor in the Department of Psychiatry and biobehavioral laboratory director in the Center for Interdisciplinary Research on Nicotine Addiction (CIRNA), formerly the Transdisciplinary Tobacco Use Research Center (TTURC). He will work closely with the Overall PI, Dr. Lerman, to ensure all study procedures and properly executed. He will oversee operations for the study and will have responsibility for the design, implementation and evaluation of the proposed research. He will be responsible for overseeing the study materials for institutional review board approval, programming study equipment, overseeing training, conducting analyses and manuscript preparation.

Caryn Lerman, Ph.D., Overall Center Principal Investigator, Dr. Lerman is Mary W. Calkins Professor in the Department of Psychiatry and Annenberg Public Policy Center at the University of Pennsylvania, and Scientific Director of the Abramson Cancer Center. She has extensive expertise leading pharmacogenetic clinical trials for the treatment of nicotine dependence, including multi-institutional trials. Dr. Lerman led the clinical PGx trials of the NMR which provide the foundation for the proposed trial. She also has extensive experience conducting human laboratory studies that clarify the mechanisms underlying genetic associations with smoking cessation.

Rachel Tyndale, Ph.D. (U. Toronto) is the Canada Research Chair in Pharmacogenetics, a Professor in the Department of Pharmacology at the University of Toronto, and Head of Pharmacogenetics for the Centre for Addiction and Mental Health (CAMH). Dr. Tyndale serves as the Co-PI for the parent current grant. She has expertise in pharmacogenetics of drug dependence with a particular focus on CYP drug-metabolizing enzymes. Her group also has expertise in animal models of metabolism in the brain and liver and their impact on drug response and behaviors. Dr. Tyndale serves as Co-Chair of the PGRN publications committee and member of the PGRN coordinating committee.

Neal Benowitz, M.D. (UCSF) is Professor of Medicine and Chief of the Division of Clinical Pharmacology at the University of California San Francisco. Dr. Benowitz has published more than 300 papers on various

aspects of nicotine pharmacology, including addiction, metabolism and kinetics, and genetic, sex and racial influences on nicotine metabolism and smoking behavior, and biomarkers of nicotine exposure.

Frank Leone, M.D., Study Physician, Dr. Leone, M.D., is an Associate Professor of Pulmonology at Penn Presbyterian, and leads a smoking cessation clinic there. He has served as Study Physician on several projects in the CIRNA, including several of Dr. Strasser's. Dr. Leone will be available throughout the study to review any reported events that may arise amongst the participant sample.

Christopher Jepson, Ph.D., Biostatistician, Dr. Jepson is a Biostatistician in the CIRNA. He has served as biostatistician on several federally-funded smoking cessation and smoking-related trials. He will provide expertise in statistical considerations, primarily data analytic strategies for biobehavioral data, addressing missing data issues, and interpreting complex behavioral outcome analyses. He has been a part of the CIRNA/TTURC team for the past 9 years. In addition, Dr. Jepson will assist in manuscript preparation.

Staff Training

Staff training will consist of an explanation of the protocol and review of all study forms and measures. In addition, the duties of each staff person will be outlined and all applicable regulations will be reviewed. All questions will be answered. Senior personnel will supervise junior staff and provide re-training in the study protocol as needed.

The following research staff will be directly involved with the implementation and execution of the current study.
Angela Pinto, M.B.A., Project Manager
Sean Fleming, B.S., Samples Manager
Kathy Z. Tang, Research Staff
Rachel Dumont, Research Staff
Paul M. Sanborn, Research Staff
Dominique Vaughn, Research Staff
Susan Ware, Database Manager

Study Facilities

The Biobehavioral Smoking Laboratory (BBSL) is located in the CIRNA and includes 4 smoking- approved, industrial-ventilated, rooms attached to a common observation and preparation room, as well as -80C and - 20C freezers. The BBSL has been used in several smoking studies from Drs. Lerman and Strasser, R01:120594, assessing over 12,000 topography smoked-cigarettes since 2005.

REFERENCES

1. Strasser AA, Ashare RL, Kozlowski LT, Pickworth WB. The effect of filter vent blocking and smoking topography on carbon monoxide levels in smokers. Pharmacol Biochem Behav. 2005;82(2):320-9.
2. Strasser AA, Lerman C, Sanborn PM, Pickworth WB, Feldman EA. New lower nicotine cigarettes can produce compensatory smoking and increased carbon monoxide exposure. Drug Alcohol Depend. 2007;86(2-3):294-300.
3. Strasser AA, O'Connor RJ, Mooney ME, Wileyto EP. Digital image analysis of cigarette filter stains as an indicator of compensatory smoking. Cancer Epidemiol Biomarkers Prev. 2006;15(12):2565-9.
4. IOM (National Research Council) Committee on Scientific Standards for Studies on Modified Risk Tobacco Products Board on Population Health and Public Health Practice. Institute of Medicine of the National Academies. Scientific Standards for Studies on Modified Risk Tobacco Products. The National Academies Press, Washington, DC. 2012.
5. Strasser AA, Benowitz NL, Pinto AG, Tang KZ, Hecht SS, Carmella SG, et al. Nicotine metabolite ratio predicts smoking topography and carcinogen biomarker level. Cancer Epidemiol Biomarkers Prev. 2011;20(2):234-8.
6. Strasser AA, Malaiyandi V, Hoffmann E, Tyndale RF, Lerman C. An association of CYP2A6 genotype and smoking topography. Nicotine Tob Res. 2007;9(4):511-8.
7. Ho MK, Mwenifumbo JC, Al Koudsi N, Okuyemi KS, Ahluwalia JS, Benowitz NL, et al. Association of nicotine metabolite ratio and CYP2A6 genotype with smoking cessation treatment in African-American light smokers. Clin Pharmacol Ther. 2009;85(6):635-43.
8. Lerman C, Jepson C, Wileyto EP, Patterson F, Schnoll R, Mroziewicz M, et al. Genetic variation in nicotine metabolism predicts the efficacy of extended-duration transdermal nicotine therapy. Clin Pharmacol Ther. 2010;87(5):553-7.
9. Lerman C, Tyndale R, Patterson F, Wileyto EP, Shields PG, Pinto A, et al. Nicotine metabolite ratio predicts efficacy of transdermal nicotine for smoking cessation. Clin Pharmacol Ther. 2006;79(6):600-8.
10. Patterson F, Schnoll RA, Wileyto EP, Pinto A, Epstein LH, Shields PG, et al. Toward personalized therapy for smoking cessation: a randomized placebo-controlled trial of bupropion. Clin Pharmacol Ther. 2008;84(3):320-5.
11. Ray R, Tyndale RF, Lerman C. Nicotine dependence pharmacogenetics: role of genetic variation in nicotine-metabolizing enzymes. J Neurogenet. 2009;23(3):252-61.
12. Federal Trade Commission Report. Tar, nicotine, and carbon monoxide of the smoke of 1294 varieties of domestic cigarettes for the year 1998. 2000.
13. Hecht SS, Murphy SE, Carmella SG, Li S, Jensen J, Le C, et al. Similar uptake of lung carcinogens by smokers of regular, light, and ultralight cigarettes. Cancer Epidemiol Biomarkers Prev. 2005;14(3):693-8.
14. Strasser AA, Tang KZ, Sanborn PM, Zhou JY, Kozlowski LT. Behavioral filter vent blocking on the first cigarette of the day predicts which smokers of light cigarettes will increase smoke exposure from blocked vents. Exp Clin Psychopharmacol. 2009;17(6):405-12.
15. National Cancer Institute. "Light" Cigaretes and Cancer Risk. National Cancer Institute Fact Sheet 10.17. U.S. National Institutes of Health, reviewed 2010 Oct (http://222.cancer.gov/cancertopics/factsheet/Tobacco/light-cigarettes). 2004.
16. U.S. Department of Health and Human Services (USDHHS) The health consequences of smoking: A report of the Surgeon General. Centers for Disease Control and Prevention, Atlanta, GA. 2004.
17. Warner KE. Tobacco harm reduction: promise and perils. Nicotine Tob Res. 2002;4 Suppl 2:S61-71.
18. Philip Morris Company. A new cigarette. A new way to smoke. Accord. Richmond, VA. 1998.
19. RJ Reynolds Tobacco Company. RJ Reynolds tobacco’s risk-reduction methodology demonstrates Eclipse cigarettes may present less risk. Press Release. Winston-Salem, NC. 2000.
20. Benowitz NL. Clinical pharmacology of nicotine: implications for understanding, preventing, and treating tobacco addiction. Clin Pharmacol Ther. 2008;83(4):531-41.
21. Benowitz NL, Lessov-Schlaggar CN, Swan GE. Genetic influences in the variation in renal clearance of nicotine and cotinine. Clin Pharmacol Ther. 2008;84(2):243-7.
22. Swan GE, Lessov-Schlaggar CN, Bergen AW, He Y, Tyndale RF, Benowitz NL. Genetic and

environmental influences on the ratio of 3'hydroxycotinine to cotinine in plasma and urine. Pharmacogenet Genomics. 2009;19(5):388-98.
23. Dempsey D, Tutka P, Jacob P, 3rd, Allen F, Schoedel K, Tyndale RF, et al. Nicotine metabolite ratio as an index of cytochrome P450 2A6 metabolic activity. Clin Pharmacol Ther. 2004;76(1):64-72.
24. Pianezza ML, Sellers EM, Tyndale RF. Nicotine metabolism defect reduces smoking. Nature. 1998;393(6687):750.
25. Mwenifumbo JC, Al Koudsi N, Ho MK, Zhou Q, Hoffmann EB, Sellers EM, et al. Novel and established CYP2A6 alleles impair in vivo nicotine metabolism in a population of Black African descent. Hum Mutat. 2008;29(5):679-88.
26. Mwenifumbo JC, Tyndale RF. Molecular genetics of nicotine metabolism. Handb Exp Pharmacol. 2009(192):235-59.
27. Schoedel KA, Hoffmann EB, Rao Y, Sellers EM, Tyndale RF. Ethnic variation in CYP2A6 and association of genetically slow nicotine metabolism and smoking in adult Caucasians. Pharmacogenetics. 2004;14(9):615-26.
28. Benowitz NL, Pomerleau OF, Pomerleau CS, Jacob P, 3rd. Nicotine metabolite ratio as a predictor of cigarette consumption. Nicotine Tob Res. 2003;5(5):621-4.
29. Malaiyandi V, Lerman C, Benowitz NL, Jepson C, Patterson F, Tyndale RF. Impact of CYP2A6 genotype on pretreatment smoking behaviour and nicotine levels from and usage of nicotine replacement therapy. Mol Psychiatry. 2006;11(4):400-9.
30. Wassenaar CA, Dong Q, Wei Q, Amos CI, Spitz MR, Tyndale RF. Relationship between CYP2A6 and CHRNA5-CHRNA3-CHRNB4 variation and smoking behaviors and lung cancer risk. J Natl Cancer Inst. 2011;103(17):1342-6.
31. Benowitz NL, Lessov-Schlaggar CN, Swan GE, Jacob P, 3rd. Female sex and oral contraceptive use accelerate nicotine metabolism. Clin Pharmacol Ther. 2006;79(5):480-8.
32. Mooney ME, Li ZZ, Murphy SE, Pentel PR, Le C, Hatsukami DK. Stability of the nicotine metabolite ratio in ad libitum and reducing smokers. Cancer Epidemiol Biomarkers Prev. 2008;17(6):1396-400.
33. United States Department of Agriculture (USDA). Tobacco Outlook, Economic Research Services, TBS-263, October 2007 (http://www.ers.usda.gov) 2007.
34. Kozlowski LT, Dollar KM, Giovino GA. Cigar/cigarillo surveillance: limitations of the U.S. Department of Agriculture system. Am J Prev Med. 2008;34(5):424-6.
35. Campaign for Tobacco Free Kids. How to make state cigar tax rates fair and effective. February 10, 2010. (Last accessed June 29, 2010: http://www.tobaccofreekids.org/research/factsheets/pdf/0335.pdf). 2010.
36. Tobacco Retailer. Small cigars are big business. Tobacco Retailer, September/October. 2011.
37. Morris DS. Tobacco manufacturing data demonstrate industry product switching in response to tax increases. Tob Control. 2010;19(5):421.
38. Dollar KM, Mix JM, Kozlowski LT. Little cigars, big cigars: omissions and commissions of harm and harm reduction information on the Internet. Nicotine Tob Res. 2008;10(5):819-26.
39. Strasser AA, Orom H, Tang KZ, Dumont RL, Cappella JN, Kozlowski LT. Graphic-enhanced information improves perceived risks of cigar smoking. Addict Behav. 2011;36(8):865-9.
40. Baker F, Ainsworth SR, Dye JT, Crammer C, Thun MJ, Hoffmann D, et al. Health risks associated with cigar smoking. JAMA. 2000;284(6):735-40.
41. Shanks TG, Burns DM. Disease consequences of cigar smoking. In DR Shopland, DM Burns, D Hoffman, KM Cummings, RH Amacher (eds.). Cigars: health effects and trends. Smoking and tobacco control. Monograph 9, 1998: 105-160. U.S. Department of Health and Human Services, National Institutes of
Health, Bethesda, MD, Publication No. 98-4302. 1998.
42. Shopland DR, Burns DM, Hoffmann D, Cummings KM, Amacher RH. (eds.). Cigars: health effects and trends. Smoking and tobacco control. Monograph 9, 1998: 105-160. U.S. Department of Health and Human Services, National Institutes of Health, Bethesda, MD. 1998.
43. Boffetta P, Nyberg F, Agudo A, Benhamou E, Jockel KH, Kreuzer M, et al. Risk of lung cancer from exposure to environmental tobacco smoke from cigars, cigarillos and pipes. Int J Cancer. 1999;83(6):805-6.
44. Henningfield JE, Hariharan M, Kozlowski LT. Nicotine content and health risks of cigars. JAMA. 1996;276(23):1857-8.

45. Rao Y, Hoffmann E, Zia M, Bodin L, Zeman M, Sellers EM, et al. Duplications and defects in the CYP2A6 gene: identification, genotyping, and in vivo effects on smoking. Mol Pharmacol. 2000;58(4):747-55.
46. Schnoll RA, Patterson F, Wileyto EP, Tyndale RF, Benowitz N, Lerman C. Nicotine metabolic rate predicts successful smoking cessation with transdermal nicotine: a validation study. Pharmacol Biochem Behav. 2009;92(1):6-11.
47. Benowitz NL, Hall SM, Stewart S, Wilson M, Dempsey D, Jacob P, 3rd. Nicotine and carcinogen exposure with smoking of progressively reduced nicotine content cigarette. Cancer Epidemiol Biomarkers Prev. 2007;16(11):2479-85.
48. Benowitz NL, Dains KM, Hall SM, Stewart S, Wilson M, Dempsey D, et al. Progressive commercial cigarette yield reduction: biochemical exposure and behavioral assessment. Cancer Epidemiol Biomarkers Prev. 2009;18(3):876-83.
49. Benowitz NL, Jacob P, 3rd, Bernert JT, Wilson M, Wang L, Allen F, et al. Carcinogen exposure during short-term switching from regular to "light" cigarettes. Cancer Epidemiol Biomarkers Prev. 2005;14(6):1376-83.
50. Howard LA, Ahluwalia JS, Lin SK, Sellers EM, Tyndale RF. CYP2E1*1D regulatory polymorphism: association with alcohol and nicotine dependence. Pharmacogenetics. 2003;13(6):321-8.
51. Tyndale RF, Droll KP, Sellers EM. Genetically deficient CYP2D6 metabolism provides protection against oral opiate dependence. Pharmacogenetics. 1997;7(5):375-9.
52. Tyndale RF, Payne JI, Gerber AL, Sipe JC. The fatty acid amide hydrolase C385A (P129T) missense variant in cannabis users: studies of drug use and dependence in Caucasians. Am J Med Genet B Neuropsychiatr Genet. 2007;144B(5):660-6.
53. Lee AM, Jepson C, Hoffmann E, Epstein L, Hawk LW, Lerman C, et al. CYP2B6 genotype alters abstinence rates in a bupropion smoking cessation trial. Biol Psychiatry. 2007;62(6):635-41.
54. Audrain-McGovern J, Al Koudsi N, Rodriguez D, Wileyto EP, Shields PG, Tyndale RF. The role of CYP2A6 in the emergence of nicotine dependence in adolescents. Pediatrics. 2007;119(1):e264-74.
55. Benowitz NL, Swan GE, Jacob P, 3rd, Lessov-Schlaggar CN, Tyndale RF. CYP2A6 genotype and the metabolism and disposition kinetics of nicotine. Clinical pharmacology and therapeutics. 2006;80(5):457-67.
56. Mwenifumbo JC, Sellers EM, Tyndale RF. Nicotine metabolism and CYP2A6 activity in a population of black African descent: impact of gender and light smoking. Drug Alcohol Depend. 2007;89(1):24-33.
57. Damaj MI, Siu EC, Sellers EM, Tyndale RF, Martin BR. Inhibition of nicotine metabolism by methoxysalen: Pharmacokinetic and pharmacological studies in mice. J Pharmacol Exp Ther. 2007;320(1):250-7.
58. Miksys S, Tyndale RF. Nicotine induces brain CYP enzymes: relevance to Parkinson's disease. J Neural Transm Suppl. 2006(70):177-80.
59. Miksys S, Tyndale RF. Brain drug-metabolizing cytochrome P450 enzymes are active in vivo, demonstrated by mechanism-based enzyme inhibition. Neuropsychopharmacology. 2009;34(3):634-40.
60. Shram MJ, Siu EC, Li Z, Tyndale RF, Le AD. Interactions between age and the aversive effects of nicotine withdrawal under mecamylamine-precipitated and spontaneous conditions in male Wistar rats. Psychopharmacology (Berl). 2008;198(2):181-90.
61. Siu EC, Tyndale RF. Selegiline is a mechanism-based inactivator of CYP2A6 inhibiting nicotine metabolism in humans and mice. J Pharmacol Exp Ther. 2008;324(3):992-9.
62. Hukkanen J, Jacob P, 3rd, Benowitz NL. Metabolism and disposition kinetics of nicotine. Pharmacol Rev. 2005;57(1):79-115.
63. Benowitz NL, Dains KM, Dempsey D, Havel C, Wilson M, Jacob P, 3rd. Urine menthol as a biomarker of mentholated cigarette smoking. Cancer Epidemiol Biomarkers Prev. 2010;19(12):3013-9.
64. Benowitz NL, Jacob P, 3rd, Fong I, Gupta S. Nicotine metabolic profile in man: comparison of cigarette smoking and transdermal nicotine. J Pharmacol Exp Ther. 1994;268(1):296-303.
65. Kandel DB, Hu MC, Schaffran C, Udry JR, Benowitz NL. Urine nicotine metabolites and smoking behavior in a multiracial/multiethnic national sample of young adults. Am J Epidemiol. 2007;165(8):901-10.
66. Swan GE, Benowitz NL, Lessov CN, Jacob P, 3rd, Tyndale RF, Wilhelmsen K. Nicotine metabolism: the impact of CYP2A6 on estimates of additive genetic influence. Pharmacogenet Genomics. 2005;15(2):115-25.

67. Strasser AA, Tang KZ, Tuller MD, Cappella JN. PREP advertisement features affect smokers' beliefs regarding potential harm. Tob Control. 2008;17 Suppl 1:i32-8.
68. Henningfield JE, Benowitz NL, Connolly GN, Davis RM, Gray N, Myers ML, et al. Reducing tobacco addiction through tobacco product regulation. Tob Control. 2004;13(2):132-5.
69. Wall Street Journal. Close, and it is a cigar. Tobacco manufacturers are accused of exploiting a tax loophole to boost sales, September 23, 2010.
70. Brown R, Burgess E, Sales S, Whiteley J. Reliability and validity of a smoking timeline follow-back interview. Psychol Addict Behav. 1998;12:101-12.
71. Fabian LA, Canlas LL, Potts J, Pickworth WB. Ad lib Smoking of Black & Mild Cigarillos and Cigarettes. Nicotine Tob Res. 2011.
72. Djordjevic MV, Stellman SD, Zang E. Doses of nicotine and lung carcinogens delivered to cigarette smokers. J Natl Cancer Inst. 2000;92(2):106-11.
73. Hecht SS, Carmella SG, Chen M, Dor Koch JF, Miller AT, Murphy SE, et al. Quantitation of urinary metabolites of a tobacco-specific lung carcinogen after smoking cessation. Cancer Res. 1999;59(3):590-6.
74. Harris JE. Incomplete compensation does not imply reduced harm: yields of 40 smoke toxicants per milligram nicotine in regular filter versus low-tar cigarettes in the 1999 Massachusetts Benchmark Study. Nicotine Tob Res. 2004;6(5):797-807.
75. Zacny JP, Stitzer ML, Yingling JE. Cigarette filter vent blocking: effects on smoking topography and carbon monoxide exposure. Pharmacol Biochem Behav. 1986;25(6):1245-52.
76. Sweeney CT, Kozlowski LT. Blocking filter vents increases carbon monoxide levels from ultralight, but not light cigarettes. Pharmacol Biochem Behav. 1998;59(3):767-73.
77. Heatherton TF, Kozlowski LT, Frecker RC, Fagerstrom KO. The Fagerstrom Test for Nicotine Dependence: a revision of the Fagerstrom Tolerance Questionnaire. Br J Addict. 1991;86(9):1119-27.
78. Pomerleau CS, Carton SM, Lutzke ML, Flessland KA, Pomerleau OF. Reliability of the Fagerstrom Tolerance Questionnaire and the Fagerstrom Test for Nicotine Dependence. Addict Behav. 1994;19(1):33-9.
79. Tiffany ST, Drobes DJ. The development and initial validation of a questionnaire on smoking urges. Br J Addict. 1991;86(11):1467-76.
80. Hughes JR, Hatsukami DK, Pickens RW, Svikis DS. Consistency of the tobacco withdrawal syndrome. Addict Behav. 1984;9(4):409-12.
81. Lee EM, Malson JL, Waters AJ, Moolchan ET, Pickworth WB. Smoking topography: reliability and validity in dependent smokers. Nicotine Tob Res. 2003;5(5):673-9.
82. Tabachnick BG, Fidell LS. Using multivariate statistics (4th ed.). Harper Collins, New York, NY. 2000.
83. Murphy SE, Raulinaitis V, Brown KM. Nicotine 5'-oxidation and methyl oxidation by P450 2A enzymes. Drug Metab Dispos. 2005;33(8):1166-73.
